# Differences in life projects among adolescents in situations of social vulnerability during the COVID-19 pandemic

**DOI:** 10.1186/s41155-025-00373-y

**Published:** 2025-12-08

**Authors:** André Vilela Komatsu, Alex Sandro Gomes Pessoa

**Affiliations:** 1 Universidade Tuiuti do Paraná, Curitiba, Brazil; 2 Universidade Federal de São Carlos, São Carlos, Brazil

**Keywords:** Life projects, Adolescents, Social vulnerability, Risk factors, COVID-19

## Abstract

**Background:**

One of the main developmental tasks in adolescence is preparing for adult roles, especially those related to work and family life, a process that is guided by future expectations that influence goal setting, planning, and behaviors adopted during this transition phase. However, adolescents living in situations of social vulnerability faced increased risks during the COVID-19 pandemic while having limited access to reliable information, compromising their future expectations and life projects.

**Objective:**

This study investigated the relationship between exposure to COVID-19-related risks, knowledge regarding prevention and transmission, and the commitment to life projects (LP) among adolescents in diverse vulnerable contexts.

**Methods:**

A total of 107 Brazilian adolescents from child protection institutions, public schools in high-risk areas, juvenile justice facilities, and services for homeless youth participated in the study. They completed the COVID-19 Exposure and Demographic Questionnaire, the Knowledge, Attitudes and Practices towards COVID-19 Questionnaire, and the Life Project Scale. Data were analyzed using latent profile analysis and moderation models.

**Results:**

Participants showed above-average exposure to COVID-19 compared to the general population and reported high levels of disbelief in vaccine efficacy. Life project engagement was heterogeneous across the sample, with three latent profiles identified. Greater knowledge about COVID-19 was positively associated with LP engagement, particularly among those who had lost a family member to the virus.

**Conclusion:**

Ensuring access to reliable information and psychosocial support is essential for fostering both engagement and continuity in life projects among vulnerable adolescents during health crises.

## Introduction

The COVID-19 pandemic has exacerbated inequalities and posed unprecedented challenges to public health, particularly affecting the most socially vulnerable segments of the population. Studies show that exposure to risks during critical periods, combined with uneven access to information and poor living conditions, has a significant impact on psychological well-being and the development of life plans, especially during sensitive stages of development, such as adolescence and youth (Cabral et al., 2023; Hartas, 2023; Ribeiro et al., 2023). Understanding the combined effects of exposure to risks, access to information, and living conditions is a key task in identifying factors of protection and vulnerability during youth, as well as in informing interventions that mitigate the negative effects caused by crisis situations, such as the COVID-19 pandemic (Baird et al., 2025). This knowledge can contribute to promoting resilience and greater social equity, encouraging young people to engage with long-term goals, even in the face of severe adversity.

Access to reliable information is a crucial determinant of how individuals perceive and respond to health risks. During the pandemic, disparities in access to communication and education channels shaped not only protective behaviors but also young people’s understanding of the crisis and their perceived control over the future (Al ahdab, 2021; Troiano & Nardi, 2021). In contexts of social vulnerability, restricted access to information may have increased uncertainty and hindered adolescents’ ability to integrate the pandemic experience into their life projects. Despite the extensive scientific research on the psychosocial impacts of the COVID-19 pandemic, which started shortly after social isolation measures were established, most studies were conducted through online surveys, which restricted the participation of groups with limited internet access, especially among young people from low-income families (Cabral et al., 2023; Santos & Lorenzo, 2024). The absence of these groups in the studies is concerning, since the negative effects of the pandemic tend to be magnified in contexts of greater vulnerability, where there is limited access to resources, social support, and quality educational and cultural opportunities (United Nations Children’s Fund, 2021).

In these contexts, the pandemic acted as a catalyst that intensified pre-existing inequalities and added new, specific stressors – such as prolonged school closures, uncertainty about the future, disruption of social networks, and loss of opportunities for participation and belonging – all of which directly affected adolescents’ sense of purpose and their ability to plan long-term goals (Baird et al., 2025; Gaynor & Wilson, 2020; Karaye & Horney, 2020). The experience of uncertainty and disruption to routines is known to undermine regulatory and motivational processes involved in goal setting and future orientation (Grupe & Nitschke, 2013; Peters et al., 2017). Moreover, emotional distress and experiences of loss and disconnection during the pandemic have further challenged young people’s sense of belonging and hope (Albuquerque & Santos, 2021; Weinstock et al., 2021). The combined effect of social isolation and accumulated risk conditions made it more difficult for many young people to sustain engagement with their life projects or to envision viable future pathways (Alvarenga et al., 2021; Fornell et al., 2023; Ribeiro et al., 2023; Prince et al., 2019). Therefore, it is essential to examine how various youth groups experienced the pandemic and identify the factors that most hindered their ability to plan and pursue their life projects. In particular, this study focuses on adolescents who, during the pandemic, were in different situations of social vulnerability, including those in institutional care, students in public schools located in vulnerable regions, adolescents serving socio-educational measures, and young people living on the streets. Although all these groups share conditions of social vulnerability, the forms that such vulnerability takes are heterogeneous. Differences in institutional structure, mobility, access to protection, and exposure to others’ health behaviors may result in distinct levels of exposure to COVID-19 risks among them (Bernardi, 2020; Deslandes, 2021; Fundação CASA, 2021).

The emergency measures implemented to address COVID-19 resulted in the closure of some public services and an overload of others, some of which were already operating at full capacity. In Brazil, adolescents experienced a long period of social isolation and school closures, with a lack of adequate resources for distance learning, particularly among public school students (Cabral et al., 2023). In this sense, structural inequalities have deepened existing educational and social inequalities. Studies show that the interruption of in-person classes resulted in significant learning losses, especially for low-income students, who faced greater difficulties in accessing remote education, resulting in below-target performance in traditional learning assessments conducted in Brazil (INEP, 2021; United Nations Children’s Fund, 2021). An empirical study conducted with adolescents in public schools in São Paulo identified high levels of depression and anxiety symptoms during the pandemic, which were intensified by habits related to screen time and changes in sleep patterns (Vazquez et al., 2022). These studies reveal the challenges that public school students faced during the pandemic, not only due to limited access to technology and the infrastructure necessary for distance learning, but also due to the negative effects on mental health, highlighting the impact of the already precarious living conditions of several young people.

The adversities generated by the pandemic added to the social and institutional challenges that previously existed. For adolescents in institutional care, who face significant challenges in their development, such as family separation and instability in forming emotional bonds, the period was marked by additional challenges. Among these challenges, the most notable were abrupt changes in routine, the cancellation of visits, restrictions on movement outside the shelter, and the overload of the professionals responsible for care, which negatively impacted the quality of the services provided. A national survey conducted between May and July 2020 on shelter services for children and adolescents found that, among the 1,327 services analyzed, providing care for a total of 14, 060 people, 1,075 people – including workers, children, and adolescents – became ill due to COVID-19, with 25% of this total being children and adolescents (Bernardi, 2020). In this context, Ordinance No. 59/2020 established guidelines for care in shelter services, emphasizing the importance of a balanced routine, with daily, ludic, and recreational activities, respecting the distance and well-being of those in care (BRAZIL, 2020). However, the direct and indirect effects of the pandemic on this population are still poorly understood – a search of the SciELO and Web of Science databases found no empirical studies in the Brazilian context.

Regarding institutionalization through socio-educational measures – the state’s response to adolescents who commit infractions, understood as crimes or misdemeanors under Brazilian law – this imposes a set of additional challenges on adolescents. Brazilian law, through the Statute of Children and Adolescents, aims to ensure that these measures are educational in nature, considering the potential for social reintegration of adolescents. However, the COVID-19 pandemic has brought serious challenges for adolescents serving socio-educational measures in both open and closed environments. For adolescents in open environments, restrictions on movement and social distancing measures have hindered monitoring by professionals and compromised the provision of educational activities, creating an environment of uncertainty and stress. For those in closed environments, the situation was even more complex: cancellation of visits, changes in services and hearings to online mode, increased institutional overload, and changes in recreational and educational activities hampered the development of bonds and the continuity of educational processes, aggravating the risks of psychological illness (Deslandes, 2021). Data released by the *Fundação CASA* (a center for offenders; 2021) in its statistical bulletin revealed that 13% of adolescents tested positive for COVID-19, compared to 7% of employees, both figures higher than the general population during the period, revealing the increased vulnerability in these institutional spaces.

The impact of institutionalization, combined with the challenges posed by the health crisis, can have lasting consequences for the psychosocial development of young people. International studies with groups of institutionalized individuals reveal that the COVID-19 pandemic has had a significant impact on the mental health and development of people in institutions, aggravating pre-existing conditions and increasing vulnerabilities (LeMasters et al., 2023; Sirdfield et al., 2022). Understanding the magnitude and repercussions of these effects is essential to inform evidence-based public policies and comprehensive protection practices, with the aim of developing more effective interventions to preserve the physical and mental health of adolescents, as well as promoting essential adaptive skills during youth and in the face of the often harsh realities that young people must face.

Another concerning aspect of the vulnerability intensified by the pandemic is that of children and adolescents living on the streets. Before the COVID-19 pandemic, it was estimated that approximately 70,000 children and adolescents were living in these conditions nationwide. With the onset of the pandemic, the vulnerability of this population has increased significantly, exacerbating problems such as food insecurity, lack of access to healthcare services, and exposure to violence (Municipal Secretariat for Social Assistance and Development, 2022). In addition, the pandemic has altered the profile of the homeless population, with a notable increase in the number of socially vulnerable women, children, and adolescents (Municipal Secretariat for Social Assistance and Development, 2021). Among this population, the severity of the problem for children and adolescents is striking due to the restrictions on isolation and physical distancing, compounded by the extremely poor hygiene conditions on the streets. However, the lack of official data on infection and death rates among homeless children and adolescents hinders a clear understanding of the extent of the pandemic’s impact on the health of this population, as well as the negative effects on their developmental trajectories.

One of the main developmental tasks in adolescence is preparing for adult roles, especially those related to work and family life, a process that is guided by future expectations that influence goal setting, planning, and behaviors adopted during this transition phase (Marcia, 1980; Sipsma et al., 2012). However, in contexts marked by material and symbolic deprivation, it becomes challenging to imagine what one wishes for adulthood and to understand the means to achieve these goals (Alvarenga et al., 2021). Young people in situations of social vulnerability experience significant restrictions in the process of building their life projects, since structural adversities and adversities specific to each social group compromise the development of consistent future expectations (Fornell et al., 2023; Prince et al., 2019; Raffaelli & Koller, 2005). While the design of a life project can work as a mobilizing tool, enabling adolescents to recognize their potential, reflect upon their pathway, and build strategies to project new futures, genuine autonomy is strongly compromised in these realities (Alvarenga et al., 2021; Prince et al., 2019).

Major stressors and losses experienced during the pandemic, such as the illness or death of family members, have been identified as critical factors affecting adolescents’ emotional well-being and their outlook on the future (Albuquerque & Santos, 2021; Weinstock et al., 2021). Such experiences of grief and disruption may interfere with cognitive and motivational processes involved in planning and goal setting, potentially weakening the connection between knowledge about the pandemic and engagement with life projects. However, the relationship between crisis contexts and adolescents’ ability to plan for the future is not necessarily linear or predictable. Research on meaning making and resilience shows that exposure to adversity can lead to diverse adaptations: some adolescents may experience hopelessness and disengagement, while others preserve a sense of purpose or even develop renewed motivation to pursue long-term goals (Bonanno & Mancini, 2008; Masten, 2004; Park, 2010). Moreover, cognitive-emotional processes such as temporal distancing and optimistic bias can buffer the perceived severity of crises, leading some young people to maintain idealized or unrealistic views of the future despite adverse conditions (Grupe & Nitschke, 2013; Peters et al., 2017). These patterns highlight the importance of empirically examining how adolescents in different vulnerability contexts experienced the pandemic and how their perceptions and aspirations were shaped by both structural constraints and individual adaptive processes.

In light of these scenarios, the uncertainties generated by the pandemic, compounded by the lack of support and positive role models and the various adversities faced by the most vulnerable groups, contribute to young people focusing exclusively on their immediate needs and the struggle for survival, significantly compromising the development of their life projects, limiting their future prospects, and hindering the full development of their potential. Thus, understanding the specificities and impacts of the pandemic on socially vulnerable groups – exposed to different forms of deprivation, risk, and institutional conditions – becomes essential to support public policies and interventions that promote protection, healthy development, and opportunities for the future. In this sense, the present study aimed to verify the extent to which exposure to the risks of COVID-19 and knowledge of the pandemic can influence commitment to life projects in varying groups of adolescents in situations of social vulnerability in the pandemic context. Based on this objective, the hypotheses of this study are:

- H1: The groups will differ in their level of exposure to COVID-19 risks, reflecting the distinct forms that social vulnerability assumes in each context (e.g., homelessness, institutionalization, schooling in disadvantaged areas, or confinement under socio-educational measures).
- H2: The groups differ significantly in their level of knowledge regarding the COVID-19 pandemic and its associated risks: The hypothesis is that adolescents from different groups have varying access to and understanding of information related to the pandemic, influenced by factors such as access to education and institutional resources.
- H3: The level of organization and commitment to life projects will vary significantly between groups, with lower levels expected among adolescents in situations of greater social vulnerability (e.g., those in institutional care, serving socio-educational measures, or living on the streets) compared to students in public schools.
- H4: There will be a significant association between the level of knowledge of COVID-19 and the level of commitment to life projects, with this relationship being moderated by the experience of losing a family member during the pandemic: The hypothesis is that knowledge regarding the pandemic positively influences commitment to life projects, and that this relationship is disrupted for those who have experienced significant family losses during the pandemic period.

## Method

### Participants

A total of 125 participants aged between 12 and 21 years (M = 16, SD = 1.8) from the five Brazilian macroregions were interviewed. The young people were recruited from different institutional settings, including institutional care (IC), public schools located in regions marked by social vulnerability (PS), institutions implementing socio-educational measures (SEM), and organizations working with young people living in street situations (SC). Eighteen participants were excluded from the analysis for not completing the Life Project Scale, resulting in a final sample of 107 adolescents. Table 1 presents the main sociodemographic characteristics of the participants, stratified by institutional setting.

**Table 1:** Sociodemographic characteristics of the sample

Sociodemographic variables	IC (*n* = 26)	PS (*n* = 30)	SEM (*n* = 32)	SC (*n* = 19)	Total (*n* = 107)
Age					
M (CI 95%)	15.6 (14.8, 16.4)	15.1 (14.5, 15.6)	17.4 (17.0, 17.8)	15.4 (14.6, 16.2)	16.0 (15.6, 16.3)
DP	1.9	1.5	1.2	1.7	1.8
Mdn (Min, Max)	16.5 (12.0, 18.0)	15.0 (12.0, 18.0)	17.0 (15.0, 21.0)	15.0 (12.0, 18.0)	16.0 (12.0, 21.0)
Gender					
Cis man	15 (57.7%)	7 (23.3%)	31 (96.9%)	9 (47.4%)	62 (57.9%)
Cis woman	7 (26.9%)	19 (63.3%)	1 (3.1%)	8 (42.1%)	35 (32.7%)
Trans woman	2 (7.7%)	0 (0.0%)	0 (0.0%)	0 (0.0%)	2 (1.9%)
I do not know how to answer	2 (7.7%)	1 (3.3%)	0 (0.0%)	1 (5.3%)	4 (3.7%)
I would rather not answer	0 (0.0%)	3 (10.0%)	0 (0.0%)	1 (5.3%)	4 (3.7%)
Skin color/race/ethnicity					
Asian	1 (3.8%)	1 (3.3%)	0 (0.0%)	0 (0.0%)	2 (1.9%)
White	3 (11.5%)	8 (26.7%)	6 (18.8%)	2 (10.5%)	19 (17.8%)
Native American	0 (0.0%)	0 (0.0%)	0 (0.0%)	1 (5.3%)	1 (0.9%)
Mixed	16 (61.5%)	15 (50.0%)	14 (43.8%)	7 (36.8%)	52 (48.6%)
Black	6 (23.1%)	6 (20.0%)	12 (37.5%)	9 (47.4%)	33 (30.8%)
Enrolled in school					
No	2 (12.5%)	0 (0.0%)	7 (23.3%)	7 (50.0%)	16 (19.5%)
Yes	14 (87.5%)	22 (100.0%)	23 (76.7%)	7 (50.0%)	66 (80.5%)
Education					
Complete ES	1 (3.8%)	2 (6.7%)	3 (9.4%)	2 (10.5%)	8 (7.5%)
Incomplete ES	12 (46.2%)	15 (50.0%)	14 (43.8%)	15 (78.9%)	56 (52.3%)
Complete HS	0 (0.0%)	0 (0.0%)	5 (15.6%)	0 (0.0%)	5 (4.7%)
Incomplete HS	12 (46.2%)	13 (43.3%)	8 (25.0%)	2 (10.5%)	35 (32.7%)
Does not know	1 (3.8%)	0 (0.0%)	2 (6.2%)	0 (0.0%)	3 (2.8%)

*IC *institutional care, *PS* public schools in regions marked by social vulnerability, *SEM* serving a socio-educational measure, *SC* street conditions, *ES* Elementary School, *HS* High School

### Instruments and measures

All participants responded to four instruments: (i) Questionnaire on characterization and exposure to COVID-19; (ii) Questionnaire on knowledge, attitude, and habits in relation to the COVID-19 pandemic; (iii) semi-structured interview; and (iv) Life Project Scale.

#### Exposure to COVID-19

 The six items in this questionnaire were developed to measure the level of individual exposure to risks associated with the COVID-19 pandemic. The questions assess varying aspects of participants’ experiences with the virus, such as symptoms (*1. Have you had symptoms of COVID-19?*), personal infection (*2. Have you been infected with COVID-19?*), proximity to other infected people (*3. Do you know anyone who has tested positive?*), loss of family members (*4. Have you lost any family members due to COVID-19?*), receipt of preventive guidance (*5. Have you received guidance on how to prevent becoming infected with COVID-19?*), and vaccination against COVID-19 (*6. Have you been vaccinated?*). Each item was dichotomous, with response options “Yes” (1) or “No” (0). A total score was created based on the number of affirmative responses, indicating the degree of exposure to COVID-19, with higher scores reflecting greater exposure. The degree of association between the items, measured by the Kuder-Richardson 20 coefficient, was 0.45. As an alternative to the summed-score approach, a score based on Item Response Theory (IRT) was used, employing a two-parameter model (discrimination and difficulty), allowing for the estimation of latent scores that are more sensitive to individual variations in item characteristics.

#### Knowledge regarding COVID-19

The nine items that compose this questionnaire were developed to assess individuals’ level of knowledge regarding the pandemic and its implications (Al ahdab, 2021). The questions assess whether participants understand the characteristics of COVID-19, such as clinical symptoms, similarity of symptoms to influenza, severity of cases, transmissibility of the virus, effectiveness of vaccines, and importance of social isolation: *(1) The main clinical symptoms of COVID-19 are fever*,* fatigue*,* dry cough*,* and muscle pain (myalgia)*,* (2) The symptoms of COVID-19 are similar to the common symptoms of the flu*,* (3) COVID-19 infection causes severe symptoms in all people*,* (4) People with COVID-19 can transmit the virus to others even when they have no symptoms*,* (5) COVID-19 infection can cause other serious health problems*,* (6) Although there is no proven cure for COVID-19*,* available treatments can aid recovery*,* (7) Vaccines for COVID-19 already exist*,* (8) While the population has not been vaccinated*,* social isolation has been the most effective measure against the rapid spread of the disease*,* (9) There is no scientific evidence regarding the effectiveness of the COVID-19 vaccine.* A total score was created based on the number of correct answers, indicating the level of knowledge regarding COVID-19 and its effects, with higher scores representing a better understanding of the disease, its consequences, and the necessary preventive measures. The internal consistency, estimated using the Kuder–Richardson 20 coefficient, was 0.35. As an alternative to the score obtained by simply adding up the items, a latent score was also calculated based on the IRT with a two-parameter model.

#### Life Project Scale

 The scale was designed to assess individuals’ level of clarity, organization, and commitment to their goals and future plans (Coscioni et al., 2024). Comprising eight items, this scale explores essential aspects of self-direction and motivation, including the ability to set goals, plan actions, and actively engage in the pursuit of personal achievements: (1) I know what I want for my life in the future, (2) I am investing a lot of time in actions related to my goals for the future, (3) I have clear goals for what I want to achieve in life, (4) I am striving to achieve what I want for the future, (5) I have a clear idea of the person I want to be in the future, (6) I have already started to put my plans for the future into practice, (7) I have already decided what to do with my life in the future, (8) I am engaged in activities to achieve my goals for the future. Responses were evaluated on a seven-point Likert scale, ranging from “Strongly disagree” (1) to “Strongly agree” (7). The total score, obtained by summing responses, indicates the individual’s degree of commitment and organization in relation to their life projects. Higher scores reflect greater alignment with personal goals and stronger commitment to the actions necessary to achieve them. Cronbach’s alpha for the present sample was 0.79 for the total scale, 0.76 for the identification factor, and 0.71 for the engagement factor.

Table 2 summarizes the distribution of the scores on each instrument.

**Table 2:** Descriptive measures of raw scores obtained on the instruments

Measure	Mean (95% CI)	SD	Median (Min, Max)
Exposure to COVID-19	1.8 (1.6, 1.9)	0.9	2.0 (0.0, 4.0)
Knowledge regarding COVID-19	6.5 (6.3, 6.8)	1.5	7.0 (2.0, 9.0)
Life Projects	45.8 (44.4, 47.2)	7.2	48.0 (22.0, 56.0)

### Data collection

As this is a multicenter study, the project was submitted to and approved by the Research Ethics Committees of all participating universities (CAAE [omitted]), ensuring the voluntary nature and confidentiality of the data through the signature of the Free and Informed Consent Form by the guardians and the Assent Form by the adolescents. In order to ensure standardization and methodological quality in data collection and analysis, a 30-hour remote training course was provided to the teams involved, consisting of seven theoretical meetings (15 h) and practical activities (15 h), addressing topics such as research with adolescents in vulnerability contexts, impacts of the pandemic, multiple case studies, qualitative approach, handling sensitive topics, and the use of software for qualitative analysis. Data collection was conducted in person in private rooms at the participants’ schools, in individual sessions lasting from 40 to 60 min.

### Data analysis

Participants were grouped according to their status of vulnerability and/or social exclusion in the pre-pandemic context, based on whether they were homeless (SC, *n* = 19), in institutional care (IC, *n* = 26), serving socio-educational measures (SEM, *n* = 32) or because they were students at public schools located in economically and socially vulnerable contexts (PS, *n* = 30). These four groups were then characterized and compared in relation to their level of exposure to the risks associated with COVID-19, their degree of knowledge regarding the pandemic and its risks, and the level of organization and commitment of the participants to their Life Projects. The comparison was made using robust analysis of variance (robust ANOVA; Mair & Wilcox, 2020). Post hoc tests were performed using linear contrasts of trimmed means (Linear Contrasts of Trimmed Means [lincon]; Wilcox, 2022). The same comparisons were performed for the measures obtained via item response theory, the complete results of which are available in Appendix A.

To verify the homogeneity of the groups in relation to their level of engagement with Life Projects, a latent profile analysis was performed. This clustering approach based on Gaussian mixture models (Scrucca et al., 2016) allows individuals to be regrouped based on their scale scores, regardless of their original group. Next, the proportion of each group within the identified profiles was verified. Finally, to verify whether the level of commitment to the Life Projects varies according to the level of knowledge regarding the pandemic (a variable that could increase the sense of stability/predictability of the pandemic situation) and whether this relationship is moderated by the loss of a family member during the pandemic (a stronger experience that was researched and that could potentially disrupt the organization of Life Projects) was verified using a linear regression with the following equation: LP_i_=*β*_*0*_+*β*_*1*_⋅KNOW_COVID_i_+*β*_*2*_⋅LOSS_i_+*β*_*3*_⋅(KNOW_COVID_i_×LOSS_i_)+ϵ_i_, where LP represents the score on the Life Project Scale, KNOW_COVID is the score on the COVID-19 knowledge scale, and LOSS is a binary variable indicating whether or not a family member has been lost due to COVID-19.

## Results

**Fig. 1:**
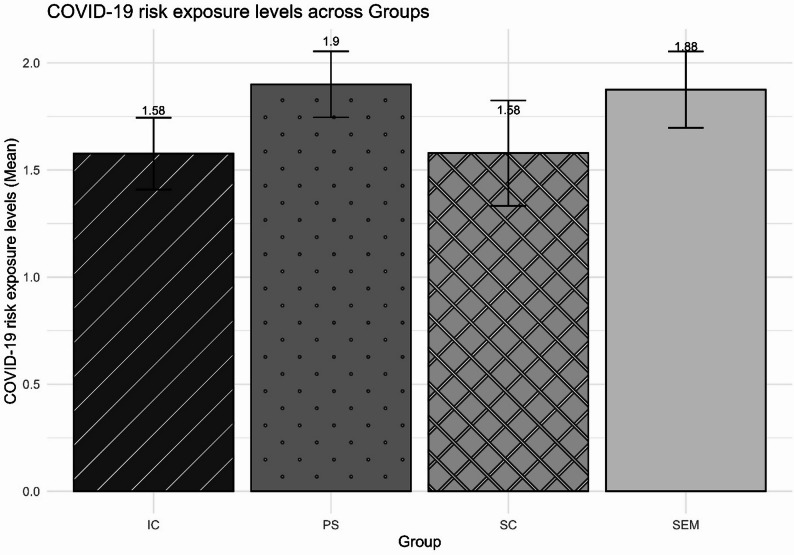
Comparison of COVID-19 risk exposure levels. Note. IC = adolescents in institutional care; PS = adolescents from public schools in socially vulnerable regions; SEM = adolescents serving socio-educational measures; SC = adolescents living in street conditions

On average, adolescents were exposed to 1.8 COVID-19 risk situations (out of 6 possible). The results indicate that, among the adolescents evaluated, most know someone who tested positive for COVID-19 (79%) and approximately half (47%) reported symptoms of COVID-19. Approximately one-quarter of adolescents have had a positive diagnosis (21%), while 20% reported losing a family member due to COVID-19. However, most adolescents received guidance on prevention (94%) and received at least one dose of the vaccine (92%). Figure 1 shows that the group means ranged from 1.58 to 1.9. Robust analysis of variance revealed no significant differences between groups based on their raw scores (*F*(3, 43.89) = 0.89; *η*^*2*^ = 0.24; *p* = 0.456), nor based on the scores obtained by the IRT (*F*(1, 3) = 0. 14; *η*^*2*^ = 0.15; *p* = 0.93).

**Fig. 2:**
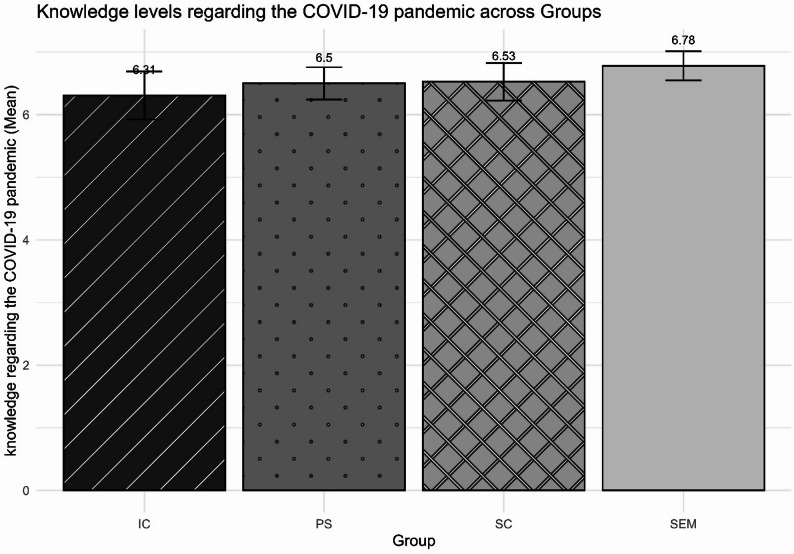
Comparison of knowledge levels regarding the COVID-19 pandemic. Note. IC = adolescents in institutional care; PS = adolescents from public schools in regions marked by social vulnerability; SEM = adolescents serving socio-educational measures; SC = adolescents living in street conditions

Regarding their level of knowledge regarding the pandemic and its effects, adolescents demonstrated correct knowledge of 6.5 out of nine pieces of information assessed. Most demonstrated knowledge of the symptoms of COVID-19 (83%) and the means of transmission (87%), but many did not believe in or were unsure of the vaccine’s effectiveness (66%). Figure 2 shows that the group means ranged from 6.31 to 6.78, with no significant differences between groups (*F*(3, 45.46) = 0.33; *η*^*2*^ = 0.24; *p* = 0.801), even when their IRT scores were considered (*F*(1, 3) = 0.14; *η*^2^ = 0.54; *p* = 0.65).

**Fig. 3:**
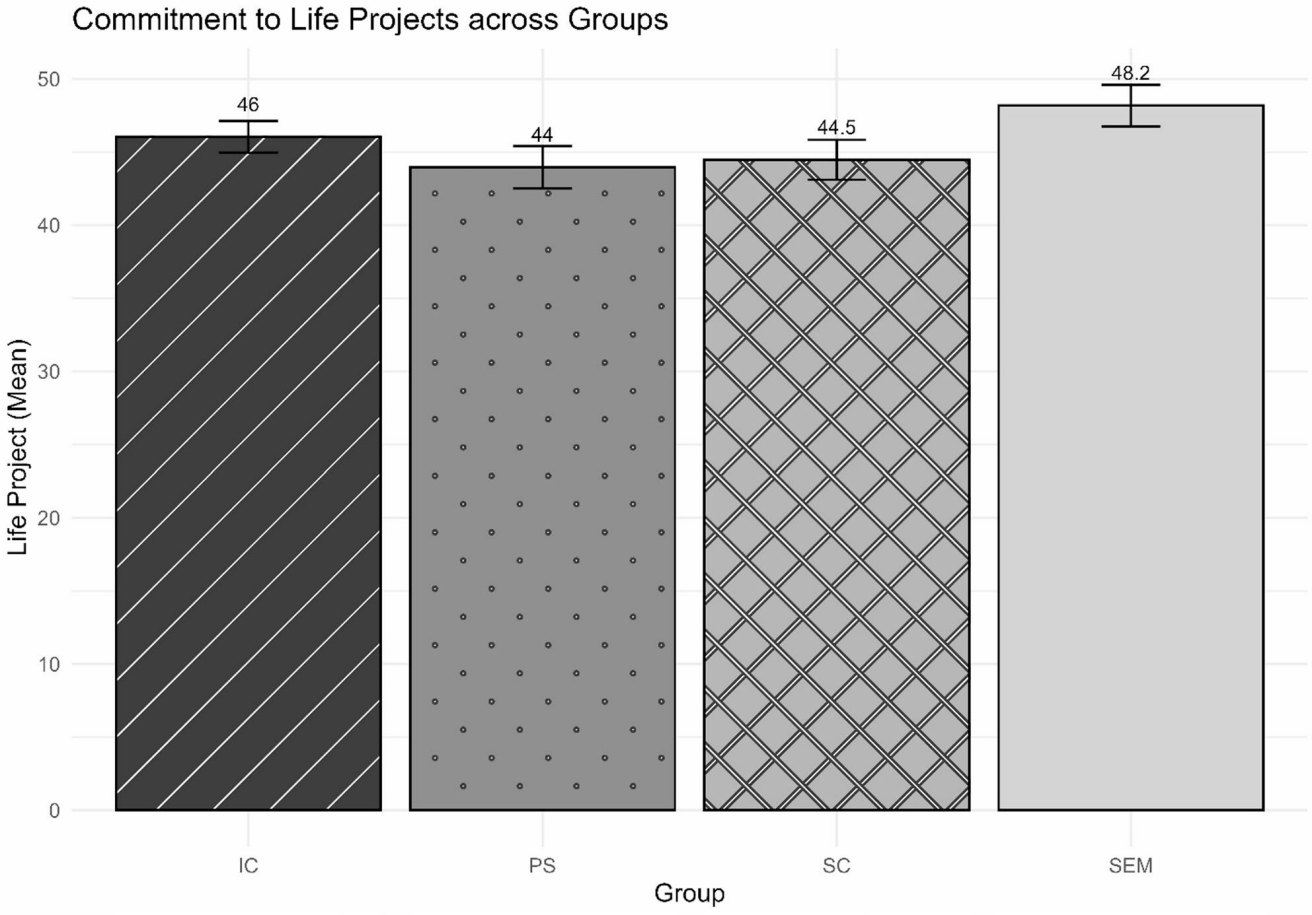
Comparison of the degree of organization and commitment to Life Projects. Note. IC = adolescents in institutional care; PS = adolescents from public schools in regions marked by social vulnerability; SEM = adolescents serving socio-educational measures; SC = adolescents living in street conditions

Figure 3, relating to the Life Project Scale, shows that the group means ranged from 44 to 49.2 (out of a maximum of 56). The analysis revealed a significant difference between the groups in terms of their level of engagement with their Life Projects (*F*(3,40.59) = 2.91; *η*^2^ = 0.36; *p* = 0.046). In particular, adolescents in SEM had a higher mean than the PS and SC groups. Furthermore, in general, the mean obtained by the sample (M = 45.8, SD = 7.22) was higher than the mean presented by the normative population (M = 42.03; SD = 8.66; *n* = 945; *p* < 0.001), even when considering only participants of a similar age group (M = 42.18; SD = 9.21; *n* = 175; *p* < 0.001).

**Fig. 4:**
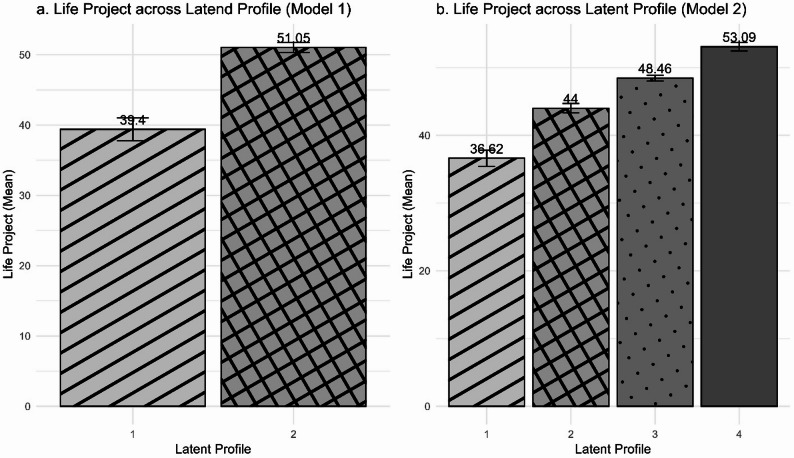
Means and confidence intervals of the Life Projects of latent profiles

Regarding the analysis of latent profiles, using the expectation-maximization algorithm, two latent profiles emerged (Fig. 4a), one with a mean score of 39 and the other with a higher mean score of 51 (out of a maximum of 56). When comparing the distribution of adolescents within the profiles, a higher ratio of public school (PS) adolescents was observed in Profile 1 (37%), representing 28% of the total sample, while the other groups (IC, SEM, SC) contributed equally with 21% each. In Profile 2, the IC, PS, SEM, and SC groups represented 27%, 20%, 37%, and 15% of the sample, respectively. However, the association between groups by institutional situation and latent profile was only marginally significant *X²*(3) = 6.1, *p* = 0.10.

Although the algorithm identified two profiles, a second analysis was performed forcing the number of profiles to four to verify to what extent they overlapped with the institutional groups (Fig. 1b). Some adjustment indices for this model were slightly higher than for model 1 (log-likelihood_1_ = −353; BIC_1_ = −730; log-likelihood_2_ = −335; BIC_2_ = −708). Profile 1, with the lowest score (M = 36.6; SD = 2.8), was composed mainly of adolescents from public schools (45%), followed by adolescents in institutional care (21%) and adolescents in socio-educational measures and street conditions, both contributing 17%. Profile 2 was the most homogeneous profile, in which each group contributed approximately 25% of the total. Profile 3 was composed mainly of adolescents in institutional care (42%), followed by adolescents in SEM (23%), SC (19%), and PS (15%). Profile 4, which had the highest scores, was mainly composed of adolescents in SEM (49%), followed by PS (24%), IC (15%), and SC (12%). The association between the groups and the profiles of model 2 was significant (*p* = 0.045). Although the four-profile model showed slightly better fit indices than the two-profile model, the difference was modest and only marginally significant. Both models are therefore presented for transparency, and interpretation focuses on the model with clearer theoretical coherence.

**Fig. 5:**
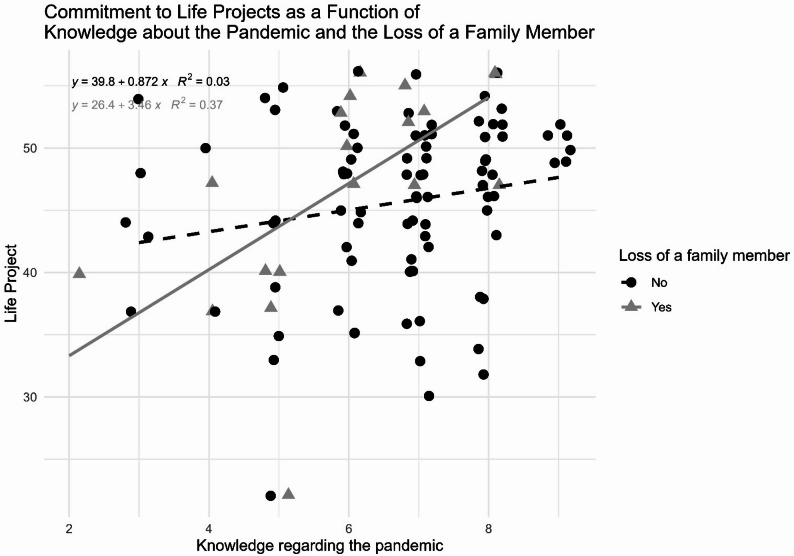
Level of identification and engagement with Life Projects based on knowledge regarding the pandemic and the loss of a family member

The hierarchical regression analysis was conducted in two steps: (1) entering exposure and knowledge variables as predictors of commitment to life projects, and (2) adding the interaction term between knowledge and family loss to test the moderation effect. Linear regression shows that the combination of the effects of knowledge regarding COVID and family loss results in an additional expected increase of approximately 2.59 on the Life Project Scale (*p* = 0.02). The results indicate two distinct linear equations, one for the group without family loss and another for the group with family loss (Fig. 5). Family loss has a substantial impact on moderating the relationship between knowledge regarding the pandemic and engagement with life projects (R² = 0.37), while the group without family loss shows an insignificant relationship (R² = 0.03).

## Discussion

The present study researched the relationship between exposure to COVID-19 risks, knowledge regarding the pandemic, and commitment to life projects among four groups of adolescents who were in socially vulnerable conditions during the pandemic. First, the adolescents were characterized separately in terms of mean scores on scales measuring exposure to COVID-19 risks, knowledge regarding the pandemic and its effects, and commitment to their life projects. Next, the homogeneity of the groups in terms of their level of engagement with Life Projects was verified using latent profile analysis. Finally, we integrated the three constructs – knowledge, risk, and Life Projects – into a regression model to verify the association between a possible protective variable (knowledge regarding the pandemic) and engagement with life projects, moderated by the risk variable of losing a family member.

Adolescents had above-average exposure to COVID-19 compared to the Brazilian population, reflected by the 47% who presented symptoms of the disease. Compared to the Brazilian population, which was approximately 28% (BRAZIL, 2024), the adolescents in this study had a 68% higher relative risk. Although national data are mostly aggregated across all age groups, available epidemiological estimates suggest that infection rates among adolescents were generally lower than among adults during the same period (Harvers, 2025). Therefore, the higher relative risk observed in our sample likely reflects not only the overall vulnerability of socially disadvantaged populations but also the cumulative effects of structural and contextual adversities experienced by these adolescents. This finding is consistent with research showing that socially vulnerable groups face greater challenges in accessing protective measures, services, and adequate health infrastructure, thus increasing their exposure to the virus and risk of infection (Gaynor & Wilson, 2020; Karaye & Horney, 2020). The fact that 20% of adolescents reported the loss of a family member due to COVID-19 reflects a distressing reality, as in addition to mourning their loss, these adolescents face the challenge of dealing with grief in an atypical way, without being able to say goodbye or experience the process in a conventional manner. The deprivation of rituals and the absence of farewells can affect the ability to process grief, especially among children and adolescents, hindering emotional regulation processes and disrupting their developmental trajectories (Albuquerque & Santos, 2021; Weinstock et al., 2021). Regarding differences between groups in terms of exposure to COVID-19, no significant differences were identified, indicating that regardless of vulnerability, the risk of infection in the groups is persistently high. Added to the social and economic instability of the period, exposure to COVID-19 is not only an immediate risk of infection but also a factor that contributes to the existential uncertainty that young people experience during this period, which can result in additional challenges to the formation of a stable identity and the pursuit of autonomy and personal achievements, such as life projects and other long-term goals. These findings highlight the need for cross-sector public policies that promote not only access to adequate health and protection services, but also broader social support that ensures the holistic development of young people.

In times of uncertainty and profound social, economic, and relational changes, such as during a pandemic, knowledge regarding the phenomenon – including routes of transmission, containment measures, coping strategies, and treatment options – acts as a protective factor by promoting a certain degree of predictability, allowing people to make safer decisions, reduce anxiety in the face of the unknown, and maintain a greater sense of control over their lives (Grupe & Nitschke, 2013; Peters et al., 2017). In this sense, we found that most of the adolescents in this study were able to recognize the main means of transmission and symptoms of COVID-19, but it is noteworthy that most of them (66%) expressed disbelief in the vaccine’s effectiveness. It is important to highlight the political context in Brazil at the time, in which political authorities and even some healthcare professionals publicly questioned international organizations such as the WHO and the effectiveness of vaccines against COVID-19 (Fleury & Fava, 2022). Official discourse often minimized the importance of immunization, generating misinformation and uncertainty among the population. Studies indicate that confidence in vaccines is influenced by sociopolitical factors and the credibility of information sources (Troiano & Nardi, 2021), which may explain the high rate of disbelief found among the teens in this study. Misinformation regarding basic aspects such as the importance of vaccines poses a significant risk to public health, potentially leading to vaccine hesitancy and the adoption of harmful behaviors. In the context of the pandemic, inaccurate or contradictory information, amplified by social media and even public figures, sought to undermine trust in science and generated confusion among the population (Fleury & Fava, 2022). For young people, who are at a crucial stage of development and decision-making regarding their future, this uncertainty can be especially harmful, generating insecurity and affecting long-term prospects. Thus, effective communication strategies are necessary to ensure that evidence-based information is accessible and understandable, helping to reduce doubts and promote informed choices.

Regarding life projects, in terms of differences between groups, adolescents serving socio-educational measures scored higher than adolescents in public schools and those living on street conditions, indicating that the support structure and resources available in each context can influence adolescents’ vision of the future. The latent profile analysis was explored using both a two-profile and a four-profile model. Although the four-profile solution presented slightly better fit indices, the difference was marginal, and both models yielded similar interpretive patterns. The four-profile model was retained for interpretive purposes because it provided a more differentiated picture of engagement patterns, even if not statistically superior. Importantly, these profiles do not correspond directly to the four institutional groups analyzed, but rather represent combinations of adolescents from different settings in varying proportions. This finding suggests that individual and contextual factors interact in complex ways that go beyond institutional boundaries. Future research could test whether specific psychosocial variables, such as self-efficacy or perceived control, contribute to the emergence of these engagement profiles.

At the same time, certain contextual characteristics may help explain the observed differences in engagement levels across institutional settings. While adolescents from public schools may experience greater stability but with fewer incentives for future planning, young people in institutional care or socio-educational measures may be more exposed to interventions that encourage the construction of new life projects. Thus, this difference may lie in the greater emphasis placed on the development of goals and future prospects in institutions that work with adolescents in conflict with the law, as part of the SINASE guidelines (BRAZIL, 2012). It is important to note that the sample had a relatively high score compared to the normative population, which was unexpected but can be explained by both contextual and psychosocial factors. It is possible that, in the face of the adversities associated with their social vulnerability, young people may develop coping strategies aimed at idealizing or actually seeking a more stable and secure future as a way to compensate for their daily challenges and establish a path to overcoming their vulnerability (Jacob et al., 2022; Masten, 2004). In this sense, it is important to emphasize the need for continuous investment in programs that strengthen support networks and offer concrete opportunities for adolescents to develop their aspirations and decide their futures with greater autonomy and agency.

Finally, by integrating three key constructs – knowledge, risk, and life projects – into a regression model to test the association between a potentially protective variable (knowledge regarding the pandemic) and engagement with life projects, moderated by a significant event (loss of a family member), this study contributes to understanding how these factors interact in a crisis context, such as the COVID-19 pandemic. The hypothesis that knowledge regarding the pandemic could provide predictability and stability amid a scenario of uncertainty and adversity, favoring greater engagement with life projects, proved significant only for those who experienced the loss of a family member. This finding suggests that significant events can intensify the search for planning and purpose, highlighting the importance of considering individual contexts and critical events when analyzing the impact of global crises on people’s lives. This interpretation resonates with theoretical perspectives that emphasize the role of meaning-making in the face of adversity, indicating that cognitive reorganization and reframing of experience can act as key mechanisms in adapting to stressful events (Bonanno & Mancini, 2008; Park, 2010). In this sense, the importance of effective communication strategies during crisis situations is highlighted, with a focus on minimizing uncertainty and facilitating adaptation, in addition to considering the interaction between individual and contextual factors in the implementation of psychosocial support programs that promote understanding and reframing of traumatic experiences, in order to reduce disruption in the significant and long-term life projects of those affected. In addition to effective communication strategies, ensuring the availability of emotional support and psychosocial care is essential to help adolescents process experiences of loss, uncertainty, and change. Such interventions can promote emotional regulation, meaning-making, and resilience, contributing to the reconstruction of a coherent sense of purpose after collective crises.

## Conclusions

This study provides original contributions and important insights into the relationships among knowledge about COVID-19, personal loss, and social vulnerability, and how these factors are associated with adolescents’ engagement with their life projects during a global health crisis. By examining multiple groups of youth in vulnerable contexts, we identified significant disparities in risk exposure and a generalized disbelief in vaccine efficacy, revealing the impact of political disinformation and systemic inequalities. Crucially, we found that knowledge about the pandemic was positively associated with commitment to life projects, particularly among those who experienced the loss of a family member, suggesting that, in the face of adversity, information and meaning making can function as stabilizing forces. These findings underscore the urgent need for cross-sector policies that not only guarantee access to reliable and context-sensitive information but also support adolescents in reframing traumatic experiences, thus sustaining engagement with future-oriented goals. By highlighting the interdependence of cognitive, emotional, and structural factors in adolescent development, this study contributes to a deeper understanding of resilience processes in crisis settings and calls for the design of psychosocial interventions that reinforce agency, continuity, and hope among youth in adversity.

However, the findings presented here should be considered in light of the study’s limitations. First, the sample consists of specific groups of adolescents, which may limit the generalization of the results to other populations, especially those outside the context of social vulnerability. In addition, the study adopted a cross-sectional design, which hinders causality analyses and limits the understanding of how the factors researched may evolve over time. The absence of variables on the institutional dynamics in which the young people were inserted also restricts the understanding of how other contextual elements could influence engagement with life projects. Furthermore, the potential influence of social desirability bias should be acknowledged, particularly among adolescents in institutional settings. Because these participants are often subject to periodic evaluations related to protective or socio-educational measures, they may perceive research participation as connected to institutional monitoring, which could lead to self-presentational responses. Although confidentiality and independence of the study were emphasized, this factor should be considered when interpreting self-reported data on motivation and engagement. Finally, the study did not address possible variations in adolescents’ experiences regarding the intensity of the loss of a family member, which could provide a more detailed understanding of the emotional and cognitive implications of this event for each adolescent. For future research, it is recommended that these aspects be considered and controlled for.

## Data Availability

The dataset generated by the survey research and analyzed for the current study are available in the OSF repository: https://osf.io/gtr8h and https://osf.io/tg68a.

## Abbreviations

COVID-19: Coronavirus Disease 2019
ES: Elementary School
HS: High School
IC: Institutional Care
INEP: Instituto Nacional de Estudos e Pesquisas Educacionais Anísio Teixeira
IRT: Item Response Theory
Lincon: Linear Contrasts of Trimmed Means
LP: Life Project
SINASE: Sistema Nacional de Atendimento Socioeducativo
SciELO: Scientific Electronic Library Online
Fundação CASA: Fundação Centro de Atendimento Socioeducativo ao Adolescente
PS: Public Schools located in regions marked by social vulnerability
SC: Street Conditions (young people living on the streets)
SEM: Structural Equation Modelling
UNICEF: United Nations Children’s Fund
WHO: World Health Organization
